# Predictive models of newborn body composition: a systematic review

**DOI:** 10.1590/1984-0462/2023/41/2020365

**Published:** 2023-03-13

**Authors:** Elissa de Oliveira Couto, Daniele Marano, Yasmin Notarbartolo di Villarosa do Amaral, Maria Elisabeth Lopes Moreira

**Affiliations:** a Instituto Nacional da Saúde da Mulher, da Criança e do Adolescente Fernandes Ferreira, Rio de Janeiro, RJ, Brazil.; b Instituto Fernandes Figueira, Rio de Janeiro, RJ, Brazil.

**Keywords:** Newborn, Regression analysis, Body composition, Recém-nascido, Análise de regressão, Composição corporal

## Abstract

**Objective:**

To analyze the prediction models of fat-free mass and fat mass of neonates who had air displacement plethysmography as a reference test.

**Data source:**

A systematic review of studies identified in the PubMed, Virtual Health Library (BVS), SciELO, and ScienceDirect databases was carried out. The Preferred Reporting Items for Systematic Reviews and Meta-Analyses (PRISMA) checklist was used for inclusion of studies, the Transparent Reporting of a Multivariable Prediction Model for Individual Prognosis or Diagnosis (TRIPOD) report was used to select only predictive models studies, and the Prediction Model Risk of Bias Assessment Tool (PROBAST) was used to assess the risk of bias in the models.

**Data synthesis:**

This study is registered in PROSPERO with identification CRD42020175048. Five hundred and three studies were found during the searches, and only four papers (six models) were eligible. Most studies (three) used the sum of different skinfolds to predict neonatal body fat and all presented weight as the variable with the highest contribution to predicting neonatal body composition. Two models that used skinfolds showed high coefficients of determination and explained, significantly, 81% of the body fat measured by air displacement plethysmography, while the models using bioimpedance did not find a significant correlation between the impedance index and the fat-free mass.

**Conclusions:**

The few studies found on this topic had numerous methodological differences. However, the subscapular skinfold was a strong predictor of neonatal body fat in three studies. It is noteworthy that such model validation studies should be carried out in the future, allowing them to be subsequently applied to the population. The development of these models with low-cost tools will contribute to better nutritional monitoring of children and could prevent complications in adulthood.

## RESUMO

**Objetivo:**

Analisar os modelos de predição da massa livre de gordura e da massa de gordura de neonatos, tendo como teste de referência a pletismografia por deslocamento de ar.

**Fontes de dados:**

Foi realizada uma revisão sistemática de estudos identificados nas bases de dados *United States National Library of Medicine* (PubMed), Biblioteca Virtual em Saúde, *Scientific Electronic Library Online* (SciELO) e ScienceDirect. O *checklist Preferred Reporting Items for Systematic Reviews and Meta-Analyses* (PRISMA) foi utilizado para a inclusão dos estudos, e o relatório *Transparent Reporting of a multivariate prediction model for Individual Prognosis or Diagnosis* (TRIPOD) foi utilizado para a seleção de apenas modelos preditivos. Para a avaliação de risco de viés dos modelos, foi empregada a ferramenta *Prediction model study Risk of Bias Assessment Tool* (PROBAST).

**Síntese dos dados:**

O presente estudo encontra-se registrado no PROSPERO com identificação CRD42020175048. Durante as buscas foram encontrados 503 estudos, dos quais apenas quatro artigos (seis modelos) foram elegíveis. A maioria dos estudos (três) utilizou a soma de diferentes dobras cutâneas para predizer a gordura corporal neonatal, e todos apresentaram o peso como a variável com maior contribuição na predição da composição corporal do recém-nascido. Dois modelos que utilizaram dobras cutâneas mostraram altos coeficientes de determinação e explicaram, significantemente, 81% da gordura corporal aferida pela pletismografia por deslocamento de ar, enquanto os modelos que utilizaram a bioimpedância não encontraram correlação significante entre o índice de impedância e a massa livre de gordura.

**Conclusões:**

Os poucos estudos encontrados sobre essa temática apresentaram inúmeras diferenças metodológicas. Todavia, observou-se em três trabalhos que a dobra cutânea subescapular é um forte preditor da gordura corporal neonatal. Destaca-se que, futuramente, devem ser realizados estudos de validação de tais modelos, permitindo que posteriormente eles sejam aplicados na população. Acredita-se que o desenvolvimento desses modelos, com ferramentas de baixo custo, trará contribuições para melhor acompanhamento nutricional infantil, podendo prevenir complicações na fase adulta.

## INTRODUCTION

Fetal development factors and neonatal growth can reflect on health throughout life.^1,2^ Studies indicate that since the pregnancy and for the next two years (for a total of about 1,000 days), a rapid growth of the newborn’s body occurs, which can permanently modify one’s biological and metabolic development and cause adaptive pathophysiological changes even in adulthood.^3,4^ Therefore, the assessment of neonatal body composition of fat-free mass (FFM) and body fat (BF) should be performed not only as a nutritional indicator, but also as a predictor of prevention of several complications in adulthood, such as chronic diseases (*diabetes mellitus*, cardiovascular diseases, cancer, chronic respiratory diseases, and neurodegenerative disorders).^5,6^

However, there is a great difficulty in clinical practice to access more accurate methods, such as dual-energy X-ray absorptiometry (DEXA), air-displacement plethysmography (ADP), or magnetic resonance imaging, mainly due to their high cost.^7,8^ Thus, more accessible measures, such as weight and length indicators, are used in neonatal nutritional assessment, composing the weight-for-age, weight-for-length, length-for-age, and the body mass index (BMI) for age indices.^8^ However, it is necessary to consider that such indicators do not evaluate components such as total body water (TBW), bone weight, BF, and FFM, which are subjected to several early childhood changes, reflecting more precisely the child’s real health.^9,10^

It is noteworthy that ADP is currently the most accurate and indicated method^11^ among the neonatal body composition assessment methods, as it does not use ionizing radiation, requires a short examination time, and allows the newborn to move without compromising the result of the test.^7,12^

Due to the difficulties in accessing the most accurate equipment in clinical practice and because isolated measures are only crude indicators of body composition, several studies have developed body composition predictive models for different age groups and audiences to enable a more accurate estimate of body composition using less expensive equipment^13,14^ over the years. It is also emphasized that selecting the desired audience for the development of the models is of paramount importance so that they are not applied in different age groups, since this variable can modify the model’s performance.^5,15^

In clinical practice, doubly indirect methods such as skinfold measurements and bioelectrical impedance analysis (BIA) are more effective, as they have a lower cost and provide evaluations of the same compartments of BF and FFM.^1,16,17^ However, models testing the correlation between these methods and with more accurate results^15,18^ must be developed to allow these measures to provide more accurate body composition data, especially neonatal. Thus, studies have developed neonatal body composition predictive models over the years through relatively low-cost and simple measures, such as skinfolds and BIA, using ADP as a benchmark method.^19,20^

Developed regarding an appropriate benchmark method for this population, these predictive models allow access to more accurate and low-cost neonatal body composition assessment in clinical practice. Therefore, this review aimed to analyze the predictive models developed for the evaluation of FFM and fat mass in newborns by assessing methodological issues of predictive neonatal BF or FFM models, which employed ADP as a reference test to understand which predictors showed a more significant correlation with neonatal body composition and which equations can be better reproduced in validation studies so that they can be performed later in clinical practice.

## METHOD

A systematic review of studies that developed a model to predict neonate body composition was carried out. These studies’ searches were carried out in PubMed, Virtual Health Library (BVS), SciELO, and Science Direct databases, with no restriction regarding language and period of publication. The advanced search tool was used for each database to select publications using keywords arranged in four blocks according to the PICO strategy. This strategy was used to select the search terms, with the guiding question being “Is it possible to predict fat-free mass or body fat using anthropometric data, skinfolds or BIA, using PDA as a reference test?”. In addition, to ensure the standardization of the selected terms, the MeSH and DeCS tools were used.

Therefore, the first block consisted of terms used for the target population of the study (newborns and infants): *“Newborn”, “Neonate”.* The second block referred to index tests: *“Anthropometry”, “Skinfold Thickness”, “Bioelectrical Impedance”.* In the third block, the term related to the comparison test used: “*Air-displacement plethysmography”;* and the fourth block, outcome, had as terms “*Fat mass*”*, “Fat-free mass”.* Boolean operators AND, OR, and NOT were used to relate the blocks to each other, aggregate at least one word from each block, and restrict the search.

Inclusion criteria for titles and abstracts for later reading of the full-text paper were based on the Transparent Reporting of a multivariable prediction model for Individual Prognosis or Diagnosis (TRIPOD).^21^ Therefore, for a paper to be included in this systematic review, we considered in the title that the study developed a model for predicting neonatal body composition, including FFM or BF. In the abstract, the information required was the description of the objectives, study design, scenario (primary and secondary care, general population), participants, sample size, predictors, statistical analysis, results, and conclusions.

Studies that assessed the prognostic factor or impact of using a predictive model in the treatment of patients, and studies exclusively evaluating the validation of existing models and whose target population was newborns diagnosed with disease or malformation were excluded.

**Figure 1.**
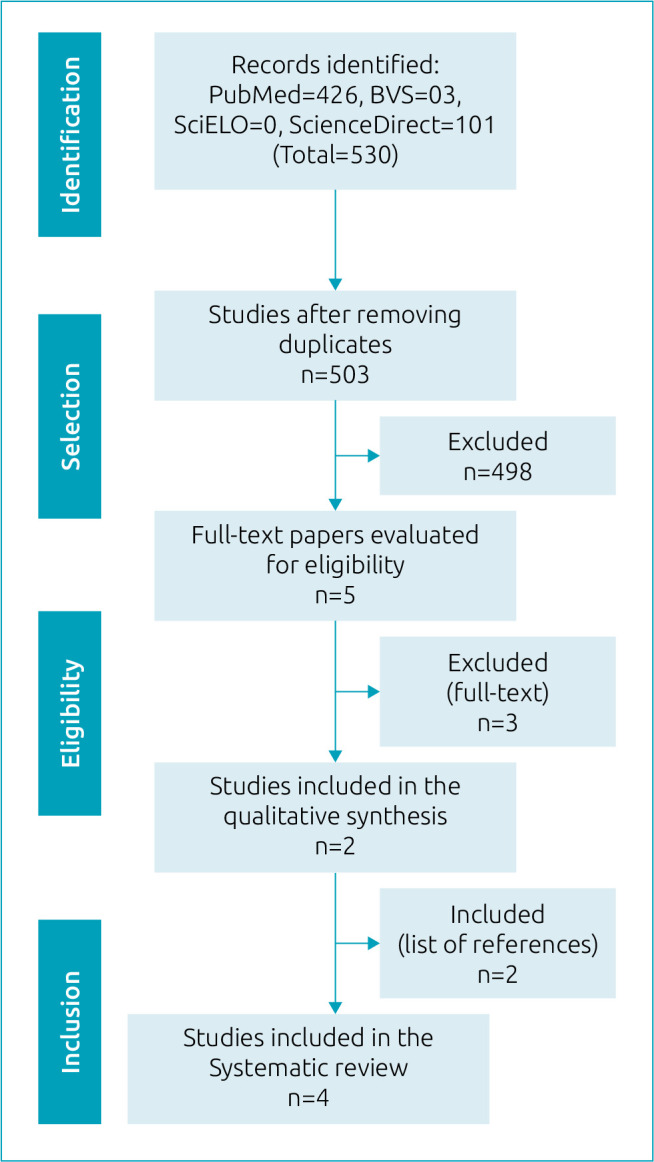
Inclusion flowchart (PRISMA).

Two independent researchers selected the titles and abstracts of the retrieved publications. Then, the full texts of the selected papers were read, checking the other steps presented in TRIPOD.^21^ Also, after reading the full texts, the search for papers that met the inclusion criteria in the reference lists was carried out, starting with reading the titles, abstracts, and full texts. The PRISMA flowchart, consisting of four stages, was used to document the number of studies considered for review according to the search strategy, identified by titles and abstracts, and included in the review after reading the full-text paper (Figure 1).

The risk of bias assessment of the included studies was carried out according to the Prediction Model Risk of Bias Assessment Tool (PROBAST),^22^ organized into four domains (participants, predictors, result, and analysis), comprising 20 questions that facilitate the structured judgment of the risk of bias in the domains mentioned above (APPENDIX 1).

The following information was extracted from the papers: author, year of publication, study location, sample size, age of newborns included, body composition predictive variables, coefficient of correlation of the development model, and standard error. This study is registered in PROSPERO with identification CRD42020175048.

## RESULTS

According to the established strategy, the search in the databases resulted in 503 studies after removing the duplicates, and of these, 498 articles were excluded, according to the criteria mentioned in the methods. Most studies were excluded because they evaluated a prognostic factor and/or the impact of the use of a prediction model in the treatment of patients; other studies were also excluded because they were either related only to the validation of existing models, or they evaluated only preterm neonates.

The five studies were then read in full, three of which were excluded for methodological reasons (two papers because they had only validated existing models and one paper with an age range outside the eligibility criteria). After checking and reading the studies’ full references, two more papers were included because they contained relevant information. Thus, this review resulted in a total of four papers for the final analysis.

Regarding the studies’ location, two were developed on the Asian continent, one in North America, and one in Oceania. The total number of newborns assessed in the four studies was 366. Most studies (three) selected term newborns (≥37 gestational weeks), and only one study found that four late preterm newborns were included (35 to <37 gestational weeks) during the development of the model. Regarding the newborns’ age, two studies excluded newborns with up to 24 hours of life, and the others included newborns from zero to four days (Table 1).^15,18–20^

**Table 1.** Data extraction from included studies.

Author, year	Country	n	Age	Variables used in the model
Deierlein et al.,^19^ 2012	USA	128	1-3 days	Sum of skinfolds (tricipital, subscapular, and thigh) gender, weight, length, age, ethnicity
Lingwood et al.,^18^ 2012	Australia	77	0-4 days	Impedance index (L^2^/R_50_), gender, weight, length or sum of skinfolds (triceps and subscapular)
Aris et al.,^20^ 2013	Singapore	88	1-3 days	Skinfold (subscapular) gender, weight, gestational age
Tint et al.,^15^ 2016	Singapore	173	0-3 days	Impedance index (L^2^/R_50_), gender, weight, length.

L: length (cm), R_50:_ resistance at 50 kHz.

Only one study developed three different predictive models, while the others developed only one model, totaling six neonatal body composition predictive models. Most models (four) used skinfold variables to predict neonatal BF, while two used BIA variables to predict FFM.

Among the studies that developed FFM predictive models, the impedance index (length squared divided by resistance at 50 kHz in ohms – L^2^/R_50_) was used. Meanwhile, studies that developed BF predictive models were different regarding the skinfolds used. The use of weight and gender variables was unanimous in all studies, while the newborn’s age, gestational age, and ethnicity were introduced in the models differently.

Only one study did not report the model determination coefficients concerning the reference test measures, and two studies did not describe the standard error value. The studies that presented these data had approximate values of the coefficients of determination and the standard error (Tables 2 and 3).^15,18–20^

**Table 2.** Body fat predictive models using skinfolds.

Reference	Equation	Skinfolds	R^2^	SE
Deierlein et al.,^19^ 2012	BF (kg)=-0.012–0.064*G+0.0024*A–0.150*W+0.055*W^2^+0.046*E+0.020*∑SF	Triceps, subscapular, and thigh	0.81	0.08 kg
Lingwood et al.,^18^ 2012	%BF_M_=1.21*∑SF–0.008∑SF^2^–1.7	Triceps, subscapular	NI	NI
	%BF_F_=1.33*∑SF–0.013∑SF^2^–2.5			
Aris et al.,^20^ 2013	BF (kg)=-0.022+0.307*W –0.077*G–0.019*GA+0.028*SF	Subscapular	0.81	NI

R^2^: coefficient of determination; SE: standard error; BF: body fat; G: gender; A: age; W: weight; E: ethnicity; SF: skin folds; NI: not informed; GA: gestational age.

**Table 3.** Fat-free mass prediction models using bioimpedance.

Reference	FFM equation	R^2^	SE
Lingwood et al.,^18^ 2012	0.822 + (0.669 * W) – (0.081 * G) + (0.016 * L^2^/R_50_)	NI	NI
Tint et al.,^15^ 2016	0.459 + (0.762 * W) – (0.045 * G) + (0.010 * L^2^/R_50_)	0.90	0.1 kg

FFM: fat-free mass; R^2^: coefficient of determination; SE: standard error; W: weight; G: gender; L: length; R_50:_ resistance at 50 kHz; NI: not informed.

According to the PROBAST, complications in the interpretation of the analyses were observed in only one study in this review, as not all the data necessary for comparison with other studies were presented. However, besides other tool stages (participants, predictors, and outcome), no study had a high risk of bias (Table 4).^15,18–20^

**Table 4.** PROBAST Result: Risk of Bias Assessment and Transparency Concerning Applicability.

Study	RB				Applicability				Overall	
	Participants	Predictors	Outcome	Analysis	Participants	Predictors	Outcome	Analysis	RB	A
1	+	+	+	+	+	+	+	+	+	+
2	+	+	+	–	+	+	+	–	+	+
3	+	+	+	+	+	+	+	+	+	+
4	+	+	+	+	+	+	+	+	+	+

PROBAST: Predictive model Risk of Bias Assessment Tool; RB: risk of bias^18^; A: applicability; + indicates a low risk of bias/low concern regarding applicability; -indicates a high risk of bias/high concern regarding applicability; and “?” indicates a clear risk of bias/unclear concern regarding applicability; 1: Deierlein et al.^19^, 2: Lingwood et al.^18^, 3: Aris et al.^20^, 4: Tint et al.^15^

## DISCUSSION

This systematic review analyzed studies that developed newborn body composition predictive models and used the ADP as a benchmark test, as recent studies confirm that this method is the gold standard for estimating BF and the FFM of newborn infants.^7,22^ Among the four papers selected for this systematic review, six different newborn body composition predictive models were analyzed. Among the studies that used BIA in the development of their models, only the results of the R_50_ were evaluated in this systematic review in order to allow comparison among studies.

Evaluation of predictive models is extremely important to determine the adiposity of newborns, since there is an increase in the prevalence of obesity in children up to five years of age.^23^ Neonatal adiposity is associated with health throughout life^1,6,24^ and may predispose newborns with greater adiposity to metabolic complications, such as type II *diabetes mellitus*, arterial hypertension, and cancer.^5,6^ Also, body composition changes in the first years of life suggest a vital role in the nutritional programming of adult morbidity.^1,25^

The assessment of body composition and weight gain is one of the main tools to understand the nutritional needs of newborns and infants.^1,11^ Based on the direct and indirect method, the assessment of body composition is impractical in clinical practice as it is expensive and depends on the evaluation of corpses.^1,23,26^

Thus, the advancement of doubly indirect methods for assessing body composition has allowed the development of new and more accurate predictive models of FFM and BF.^5,15^ Also, it is well established in the literature that birth weight, assessed in isolation, and other indices that use weight and length, such as BMI, do not adequately represent the newborn’s body fat.^11,26^

Given this matter, as mentioned, researchers reinforce that the indirect method of ADP is currently the gold standard method for assessing neonatal body composition.^11,27^ This is because ADP, measured by the PeaPod^®^ equipment, is an easy and quick application method, without exposure to radiation. It is more accurate to estimate BF and FFM from preterms (≤30 gestational weeks) to infants (six months of life or a maximum of eight kilos).^11,27^ On the other hand, it is necessary to consider that ADP is very expensive, and the equipment requires adequate space for allocation, thus resulting in difficult access in clinical practice.^1,11^ In this way, authors have developed body composition predictive models for newborns, using less expensive equipment, such as BIA and skinfold measurements, as well as information on length, weight, age, gender, ethnicity, and gestational age^15,18–20^

An essential point among the selected studies is that the newborns’ age was different in the models. Studies by Deierlein et al.^19^ and Aris et al.^20^ excluded newborns with less than 24 hours of life, justifying that rapid body changes occur in the first hours of life, which may interfere with the model’s development. The other studies^15,18^ included newborns since birth in their models.

Among the different variables introduced in each model, only the models that used the BIA were homogeneous, using weight, gender, and impedance index (L^2^/R_50_)^15,18^ for the prediction of neonatal FFM^15,18^ and the same BIA equipment model (Impedimed SFB7 – Impedimed, Brisbane, QLD, Australia). As for the results, it is essential to highlight that, although the models explained over 90% of neonatal FFM, specifically, the L^2^/R_50_ was not significant.^15,18^ Therefore, according to the authors of the evaluated studies, BIA is a limited variable in FFM’s prediction, especially in the first days (0-4 days).^15,18^

These models using BIA in newborns may not have found applicability of the methodology in the prediction of FFM because estimating FFM requires BIA to be based on the assumption that adipose tissue is essentially non-conductive. However, a vast vascular supply^28^ is found in the newborn’s adipose tissue. Also, the percentage of water in the newborn is about 45 to 48%. Thus, higher vascularization of adipose tissue and elevated water content in the first months of life can increase fat conductivity, limiting the use of BIA as a predictor of FFM.^29,30^ It stands out that, although the study of Tint et al.^15^ has not developed a specific model for BF, the authors inform that it can be estimated by subtracting the FFM calculated from body weight.

The other models used skinfolds as body composition predictors, which have a more significant correlation with BF.^18–20^ Among these models, a divergence regarding the variables tested was observed and introduced in the final model, mainly concerning the selected skinfolds. In the model developed by Deierlein et al.,^19^ four skinfolds were tested and three were introduced (subscapular, tricipital, thigh). In the final model by Aris et al.,^20^ two skinfolds were tested, and only one (subscapular) was introduced. And the models by Lingwood et al.^18^ showed no detail on how many skinfolds were tested to predict BF, but they explained that two skinfolds were included in the final model (subscapular and tricipital).

These studies were uneven regarding the correlation of some variables, mainly skinfolds, concerning BF measured by ADP. In the study by Aris et al.,^20^ while showing a high correlation with the neonatal BF measured by ADP (r=0.99), the tricipital skinfold was not significant, which according to the authors, possibly occurred due to the triplicate measurements of this fold, resulting in more significant intra-subject variability. Studies by Deierlein et al.^19^ and Lingwood et al.^18^ introduced the tricipital skinfold in the final model, but only Deierlein et al.^19^ claimed that it was significantly correlated with the BF measured by ADP (r=0.70). The model by Deierlein et al.^19^ also suggested that the thigh’s skinfold is a predictor with a highly significant correlation of BF (r=0.64).

The subscapular skinfold was the only fold with a significant correlation with the neonatal BF measured by ADP in the models by Deierlein et al.^19^ and Aris et al.^20^ (r=0.73 and r=0.99, respectively). This finding corroborates previous studies’ findings, which report that this fold represents a significant measure of central adiposity.^13,31^ These studies also described that the introduction of skinfolds, along with other variables in each model, significantly increased the coefficient of determination of BF *vis-à-vis* the benchmark method, explaining about 80% of the variability of BF measured by ADP. However, the study by Lingwood et al.^18^ pointed out that the skinfolds introduced in their model (tricipital and subscapular) conferred a high measurement bias, underestimating the percentage of neonatal fat. The authors did not discuss this finding.

The introduction of other variables in each model, such as ethnicity, neonatal age, gestational age, and gender, was also different between studies. As for the gender issue, all studies have given importance to this variable, since it is described as a determining factor in the body composition of term newborns.^32^ For this reason, the World Health Organization (WHO) recommends monitoring child growth through curves for each gender.^33^ Studies have observed that endogenous testosterone production in boys is more significant throughout the first months of life, promoting an increase in FFM, sustaining a linear growth speed.^34^

This variable was treated differently in the fat percentage prediction models of Lingwood et al.,^18^ because authors developed two different models, one for females and one for males, while the other models in this present review systematically introduced the gender variable in the equation, both for predicting BF and FFM.^15,18–20,35^

The skin color/ethnicity variable may be associated with neonatal body composition.^19,20^ Researchers suggest that the early manifestation of ethnic differences in body composition implies that this is due to genetic or maternal physiological influences.^32^ However, only the study by Deierlein et al.^19^ found that Hispanic ethnicity was a significant predictor of neonatal BF (p=0.01), and therefore considered it relevant to keep it in the model. The study by Lingwood et al.,^19^ composed of 89% of Caucasian neonates, and the study by Tint et al.,^15^ composed only of Asian newborns, did not consider this variable in the model.

Gestational age was tested only in the model by Deierlein et al.,^19^ but it was not included in the final neonatal BF prediction model, and its exclusion was not justified. Meanwhile, Aris et al.^20^ introduced a gestational age variable in the final model, since it predicted 15% of neonatal BF. Previous studies have already described that the body fat percentage of newborns increases with gestational age.^32,35^

It is important to emphasize that three of the four papers selected for this systematic review had high coefficients of determination between the developed predictive model and the ADP. Studies by Deierlein et al.^19^ and Aris et al.^20^ employed skinfolds and found the same coefficients to determine the BF obtained by the model against the reference test (R^2^=0.81). Also, Deierlein et al.^19^ described a low standard error of 0.08 kg. While they did not find BIA significance in the model, Tint et al.^15^ also found a high R^2^ of 0.90 and a low standard error of 0.1 kg. Aris et al.^20^ did not show standard error data, and Lingwood et al.^18^ did not describe the coefficient of determination and standard error.

It should be emphasized that, in clinical practice, these models with high coefficients of determination bring practicality to the assessment of neonatal body composition, allowing an early assessment of possible body changes.^15,19,20^ Its predictors are considered easy to measure and low cost,^19,20^ unlike BIA which, despite having a high coefficient of determination, in addition to not presenting statistically significant results in relation to the FFM, requires protocols for the use and calibration of the equipment.^15^

However, considering the issues mentioned above, when performing the risk analysis of bias in the studies included in this systematic review, using the PROBAST tool, it was observed that models developed by Deierlein et al.^19^ and Aris et al.^20^ had a low risk of bias and, in similar population samples, can be reproduced in model validation studies.

On the other hand, models developed by Lingwood et al.^18^ were the only ones that were of more significant concern, specifically regarding the analyses, as they did not clearly show how all the data were handled. In the study by Lingwood et al.,^18^ the results of the correlation and determination coefficients were not presented as in the other studies, considering the analysis domain with a higher risk of bias. However, when looking at the other topics to be analyzed by the PROBAST tool, we can affirm that, in this systematic review, no study evidenced a high risk of bias. Nevertheless, it is essential to emphasize that attention is required regarding the models developed by Lingwood et al.,^18^ since the model’s performance may be overestimated and different results can be found if they are reproduced in different populations.

It is interesting to point out that the studies did not focus on the discussion of protective factors, whether from BF or FFM, as they only addressed predictors and how much they contribute to the models. It is known that, in early life, both BF and FFM provide protective factors for the baby’s growth and development, preventing unfavorable health outcomes.^36^

Given the data presented, it is observed that the models included in this systematic review were able to predict the neonatal composition with high coefficients of determination, except for the study conducted by Lingwood et al.,^18^ which did not report this coefficient in any of its developed models. It is also essential to highlight the methodological disparities between these models, especially regarding the variables selected to predict neonatal body composition.

This systematic review’s results allow us to conclude that there are still few studies that have developed predictive models of neonatal FFM or BF, using ADP as the benchmark test. Furthermore, as reported in two studies, for the development of predictive body composition models of this population, care is needed in the selection of the sample when using BIA, in order to minimize possible biases. It should also be noted that it is of utmost importance to carry out more studies with the proposal to develop neonatal body composition predictive models in different populations and ethnicities, and studies to validate predictive models, thus allowing health professionals access to low-cost and high-precision nutritional diagnostic techniques in clinical practice.

## Appendix 1. PROBAST: Prediction model Risk Of Bias ASsessment Tool, for applicability.

**Table:**

	1	2	3	4
**1. Participants**				
1.1 Were appropriate data sources used, for example, cohort, RCT, or nested case–control study data?	Y	Y	Y	Y
1.2 Were all inclusions and exclusions of participants appropriate?	Y	Y	Y	PY
**2. Predictors**				
2.1 Were predictors defined and assessed in a similar way for all participants?	Y	Y	Y	Y
2.2 Were predictor assessments made without knowledge of outcome data?	NI	NI	NI	NI
2.3 Are all predictors available at the time the model is intended to be used?	Y	Y	Y	Y
**3. Outcome**				
3.1 Was the outcome determined appropriately?	Y	Y	Y	Y
3.2 Was a prespecified or standard outcome definition used?	PY	PY	PY	PY
3.3 Were predictors excluded from the outcome definition?	PY	PY	PY	PY
3.4 Was the outcome defined and determined in a similar way for all participants?	Y	Y	Y	Y
3.5 Was the outcome determined without knowledge of predictor information?	PY	PY	PY	PY
3.6 Was the time interval between predictor assessment and outcome determination appropriate?	NI	NI	NI	NI
**4. Analysis**				
4.1 Were there a reasonable number of participants with the outcome?	Y	Y	Y	Y
4.2 Were continuous and categorical predictors handled appropriately?	Y	PY	Y	Y
4.3 Were all enrolled participants included in the analysis?	Y	Y	Y	Y
4.4 Were participants with missing data handled appropriately?	NI	NI	NI	NI
4.5 Was selection of predictors based on univariable analysis avoided?	Y	Y	Y	Y
4.6 Were complexities in the data (e.g., censoring, competing risks, sampling of control participants) accounted for appropriately?	NI	NI	NI	NI
4.7 Were relevant model performance measures evaluated appropriately?	Y	NI	PY	Y
4.8 Were model overfitting and optimism in model performance accounted for?	Y	PN	Y	PY
4.9 Do predictors and their assigned weights in the final model correspond to the results from the reported multivariable analysis?	Y	PN	Y	Y

RCT = randomized controlled trial, Yes (Y), Probably yes (PY), Probably no (PN), No information (NI). 1 = Deierlein et al., 2012^19^ 2 = Ligwood et al., 2012^18^ 3 = Aris et al., 2013^20^ -4 = Tint et al., 2016^15^ (Moons et al., 2019^22^).
